# From Sputnik to Starship: Estimating the experience curve of space launch technology

**DOI:** 10.1093/pnasnexus/pgag217

**Published:** 2026-07-14

**Authors:** Alessio Terzi, Francesco Nicoli

**Affiliations:** Bennett School of Public Policy, University of Cambridge, Cambridge CB3 9DP, United Kingdom; Economics Department, Sciences Po, Paris 75337, France; Department of Management and Production Engineering, Politecnico di Torino, Turin 10129, Italy

**Keywords:** space economy, experience curves, Wright’s Law, technology forecasting, Social Sciences | Economic Sciences

## Abstract

Space launch costs have fallen dramatically over the past six decades, opening new economic and scientific frontiers. Yet precise, long-term estimates of these cost reductions remain sparse, and the underlying rate of technological learning is poorly understood. We introduce the largest standardized dataset of rocket launches to date, covering more than 4,400 launches (1960 to 2025) across 16 spacefaring geographical entities and show that the average cost of sending a kilogram to orbit has dropped from 87,023 USD in 1960 to 3,868 USD in 2025. Using a Wright's Law framework, we estimate that for each doubling of cumulative payload to orbit, the average cost of sending a kilogram to orbit decreases by 21.2%, revealing an exceptionally steep experience curve, outpacing that of other transformative technologies, including 19th-century steamship freight and modern solar photovoltaics. We leverage our empirical model to produce out-of-sample projections under a set of scenario-based and data-driven approaches. In our central estimate, the average cost is expected to fall to 1,600 USD/kg by 2030 and 300 USD/kg by 2040. Nonetheless, geopolitical shifts, potential monopolistic behavior in commercial launch markets, and the rising challenges of orbital debris may temper these gains. Overall, our findings underscore the importance of continued research and policy focus on launch technology, given that breakthroughs in cost reduction could accelerate the evolution of the space economy and its diverse near-term applications.

Significance statement Access to space has historically been constrained by high launch costs, yet the ability to analyze this constraint has been limited by fragmented data and the absence of a coherent empirical framework. This paper overcomes that barrier by assembling a comprehensive, standardized dataset of global launch costs since 1960. Using this data, it identifies a clear experience curve for launch technology and disentangles the relative importance of genuine learning by doing from shifts in market structure in driving historical cost reductions. It also provides transparent, model-based cost forecasts calibrated on the data, offering a reproducible alternative to speculative industry projections. Together, these contributions establish the empirical foundation needed to understand and evaluate the evolving accessibility of space from an economic perspective.

## Introduction

Space holds huge value from a scientific, economic, and military standpoint. Many applications first developed for space now have Earth-based applications, including air and water filtration systems, natural soil decontamination techniques, high-efficiency solar cells, electric/solid-state battery enhancements, and electric aviation technology (1, 2). Thanks to the fundamental role of satellites for information and telecommunication technologies in Low-Earth Orbit (LEO), it is estimated that the space economy was already worth over 600 USD billion in 2024: roughly as much as Sweden's gross domestic product (3). From a geopolitical point of view, much of military communication, navigation, and observation capability rely on satellites, making space a national security priority for both the United States and China (4, 5).

What has made all of this possible is the fact that the cost of accessing space has been falling substantially since humanity's first venture into space with Sputnik in 1957 (6). While this dynamic has been described theoretically or anecdotally, precise long-term estimates of the fall in the average cost of sending a kilogram of payload into orbit are patchy (7–9). Much of the literature has focused on estimating the cost of each rocket, which was of interest from an engineering standpoint but fails to take into account how often these rockets were used and therefore what the average cost of reaching space was (10–12). Others have tried to estimate the historic trend in satellite launch costs but focused on short-time horizons (13, 14).

In this paper, we are interested in estimating for the first time the long-term experience curve for space launch technology as has been done before with a variety of other technologies (15, 16). This is crucial because it will give us a sense of the exact rate at which the cost of payload to orbit has decreased over the decades. In addition, it will allow us to make informed projections about the potential for future cost reductions. If and when these reductions materialize, new applications in space will become financially viable, including, for instance, microgravity manufacturing for high-quality fiber cables or pharmaceuticals (17–19). This in turn would amplify the direct contribution of space to economic growth, going beyond the indirect innovation channel that has been quantified for space or past programs like the Apollo missions (2, 20).

To perform our quantitative analysis, we collated from a variety of academic, government, and public sources the most comprehensive standardized dataset of rocket launches from 1960 to 2025, covering 16 geographical entities, including all the major spacefaring nations: the United States, Russia, China, India, Europe, and Japan (see Materials and methods). Encompassing over 330 different rocket configurations and 4,405 launches, our dataset allows us to precisely estimate the current average costs of accessing space, how these have been evolving over the years, whether their reduction is large, and to extrapolate their likely future evolution. Crucially, the granularity of the data also allows us to separate the role of genuine learning by doing within rocket families from that of shifts in market structure in driving these cost reductions and to document how the balance between the two has changed over time.

## Results

Our data confirm the intuition that there has been a very significant reduction in the average cost of launching a kilogram of payload to orbit, and specifically in 2024 USD to LEO, which is the unit to which all our data is standardized. In our estimates, this went from over 87,000 USD/kg in 1960 to 3,868 USD/kg in 2025, indicating a reduction of over 20-fold (Fig. S1). Looking at the overall time profile, the reduction has been very fast during the rush to the Moon in the 1960s and early 1970s, going down to roughly 20,000 USD/kg. With the end of the Apollo project and the phase-out of its super-heavy Saturn V rocket, the average cost of sending a kilogram to orbit slightly increased and remained broadly stable until the early 1990s, when the fall in costs has picked up once more.

**Figure 1:**
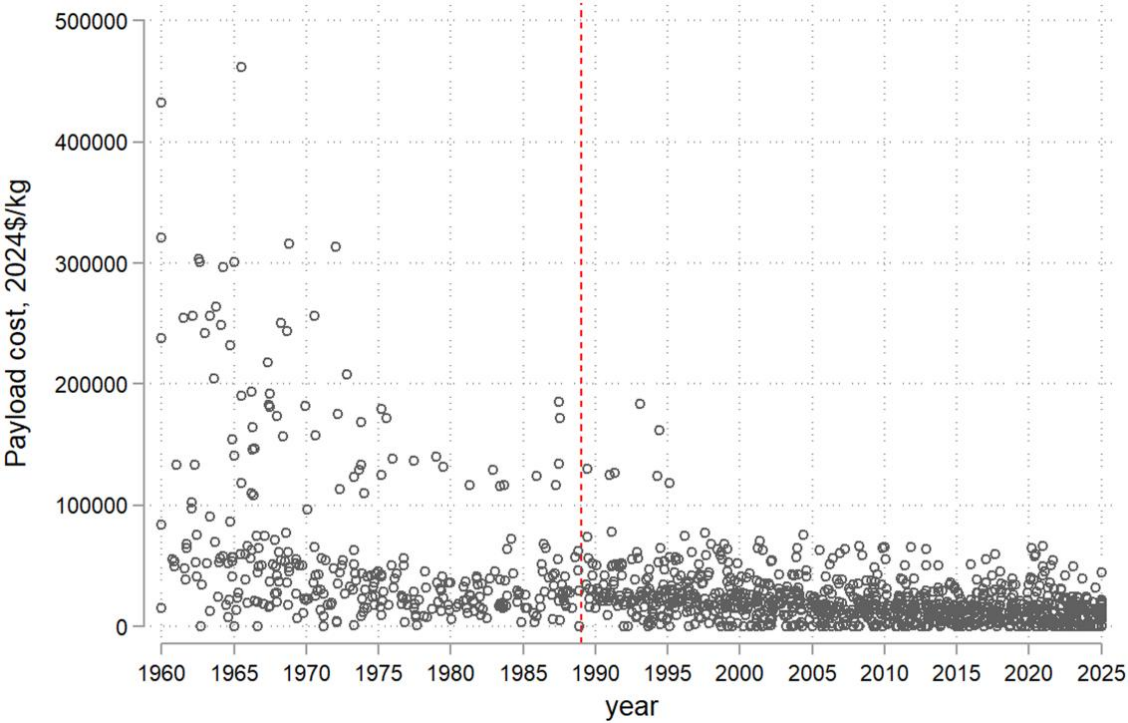
Cost per kilogram of payload to orbit, by launch. Each dot represents a single launch. Costs are expressed in 2024 USD. Dotted line marks the end of the Cold War. Chart excludes outliers (five observations with cost per kilogram above 1 million USD).

The granularity of our data allows us to go beyond averages to observe also how the dispersion of launch costs has shrunk over the years (Figs. 1 and S2). Three effects could be causing this phenomenon. First, historically it has been documented how there is a high degree of trial and error in the early application of technologies, leading to a wide dispersion in productivity outcomes (21). Second, space exploration began in a Cold War era and was dictated by the objective of showcasing scientific advancements. As a consequence, launch costs were a secondary concern, especially with respect to reliability (11, 22). Spacefaring nations would want to launch using their home-based rocket technology irrespective of the cost. Third, and relatedly, starting in the 1980s, the private sector has become interested in placing its own satellites in space, predominantly for telecommunication purposes. Evidently, this has progressively led to a shifting focus toward profitability and cost minimization.

To get a better sense of what was driving cost reductions, we performed a two-way Bennett decomposition on our data (Fig. S3). We split our sample in before and after 1995: the date where variance in launch costs stabilizes at lower levels (Fig. S2). This technique parses out the change in average launch costs due to reductions that took place within rocket families, suggesting a degree of learning by doing from those that were driven by a reallocation of payload toward other rocket families. Our data indicate that the aggregate cost decline in the early period was driven entirely by reallocation, with within-family costs actually rising. This is consistent with the fact that governments were testing many designs early on; most of these were not iterated enough to see learning by doing, and those that were selected were not getting cheaper. These stylized facts are in line with the standard life cycle of new sectors (23). In the post-1995 period, both within-family learning and reallocation contribute to cost reduction, indicating that following a shakeout in the number of manufacturers and early unsuccessful rocket designs, genuine learning by doing operated alongside competitive selection in a more mature industry. In terms of relative size, over the last three decades the scaleup of payload in cheaper rockets has been a more important contributor to average cost reduction than learning by doing within the same rocket family. We will return to this finding in the Discussion.

### Wright's Law

**Figure 2:**
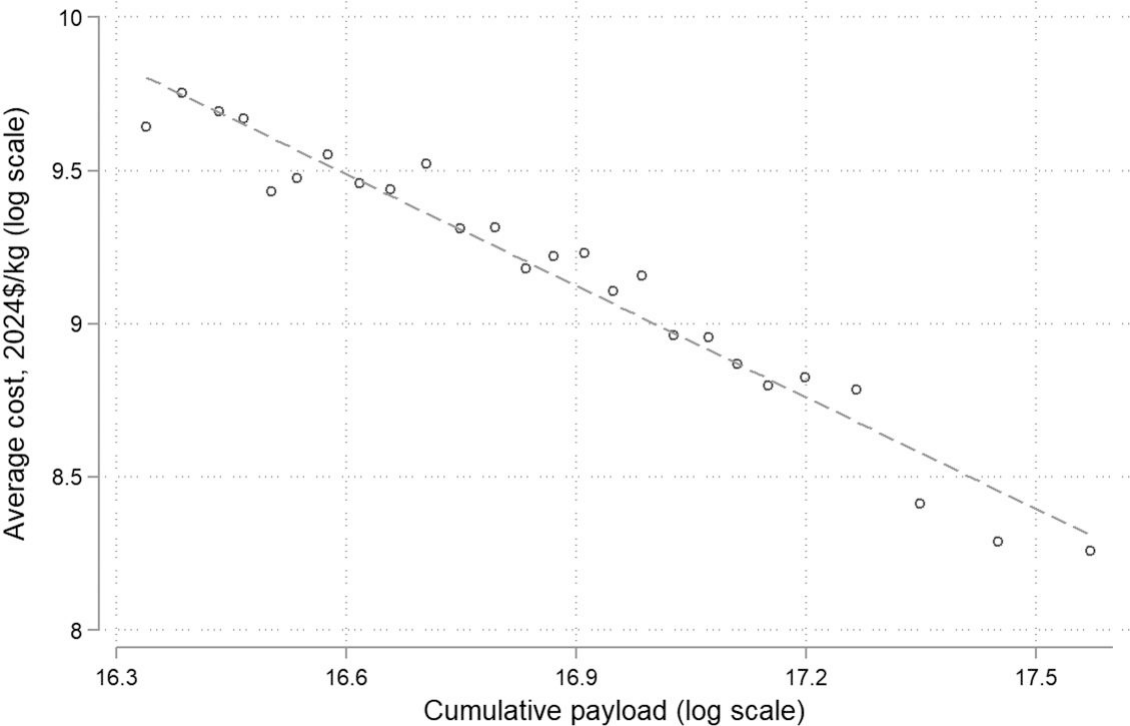
Wright's Law for space launches, 2000–2025. The *x*-axis displays the logarithm of cumulative payload to orbit. The *y*-axis displays the logarithm of the average cost of sending a kilogram of payload to LEO. The dashed gray line represents the linear best fit.

In 1936, Theodore Wright heuristically observed that many technologies display a constant rate of cost reduction, specifically as a function of cumulative production (24). More formally, he observed that


$$\log\;(c_{t})=\theta +\beta \log\;\left(\sum_{0}^{t}m_{t}\right)+\varepsilon_{t},$$

where, adapting it to our context, *c_t_* is the average launch cost in year *t*, *m_t_* is the maximal payload capacity launched in year *t* and *β* is a constant cost improvement rate. In its original formulation, this equation defined the *learning* curve of a specific technology, as cost reductions were a function of standardization and economies of scale. “Experience” curves are a broader concept that encompasses all cost reductions within a product category, including not only learning effects but also process innovation, changes in market structure, and generational leaps. It has been shown that macro-level experience curves can follow the same functional form as microlevel learning curves (15, 25). We therefore tested whether space launches follow Wright's Law. Our data show how the experience curve of space launchers follows very closely Wright's Law (*R*^2^ = 0.791), and as displayed in Fig. 2 and Table S1, this is particularly the case since the turn of the century (*R*^2^ = 0.953). Specifically, it suggests that for a 1% increase in cumulative payload sent to space, we observed on average a 0.34% fall in launch costs per kilogram. This implies that each time cumulative payload doubles, the average cost of sending a kilogram to orbit decreases by ∼21.2%. If we split our sample before and after the Cold War, we can show statistically that the rate of cost reduction has been much faster after 1989. Specifically, costs decreased by roughly 44% with each doubling of payload in the post-Cold War era, and only 17% during the Cold War. As a further check, we teased out the “learning” curve of space launches, controlling for family and year fixed effects. In all specifications, as cumulative payload within a rocket family expands, the associated average launch costs declined, at a rate of roughly 6% for each doubling of cumulative payload. Since the end of the Cold War, the learning rate accelerated to 8% (Table S2). Taken together, this is prima facie evidence that state-led competition during the Cold War did not foster cost efficiency improvements on a similar scale as those observed during the ensuing era of international space cooperation and private sector involvement.^a^

Another form of experience curve is the one identified by Gordon Moore in 1965, specifically for transistors technology. In Moore's case, the progress ratio is a fixed function of time (26). We found that Moore's Law is a slightly poorer fit for space data than Wright's Law (*R*^2^ = 0.739), and the same is true when restricting the sample to this century (*R*^2^ = 0.914). It does however suggest that the average cost of sending a kilogram of payload to orbit has fallen on average by 2.9% each year.^b^

In what follows, we show that the cost reductions observed for space launch technology are quantitatively large by comparative and historical perspective.

### Comparison to solar photovoltaic

**Figure 3:**
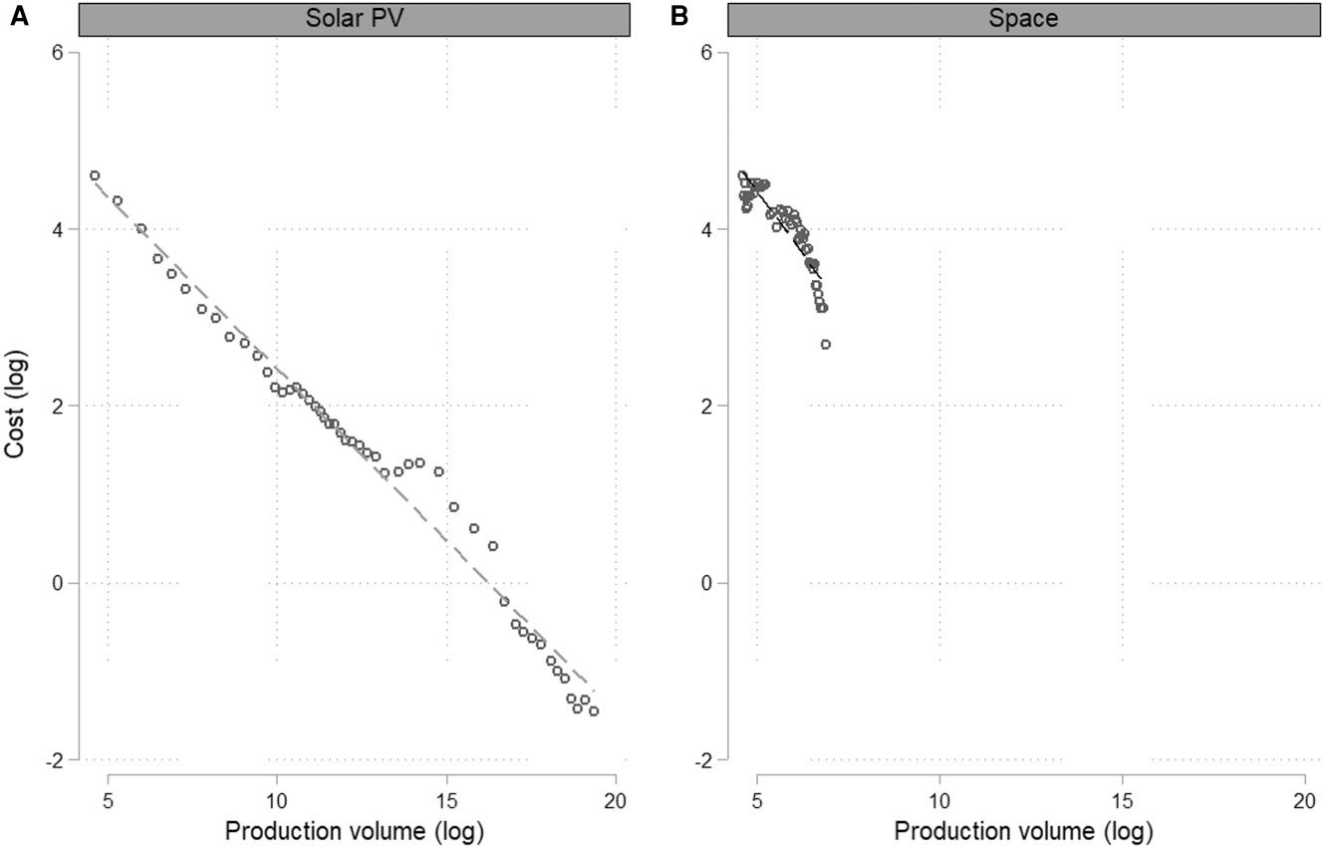
Comparison in experience curves between solar PV (A) and space launch (B), 1975–2023. The *x*-axis displays the logarithm of an index of cumulative production volume. For space, this is an index of the cumulative payload to orbit. For solar PV, this is an index of solar PV cumulative capacity. The *y*-axis displays the logarithm of an index of the average cost of solar modules and space launch per kilogram. Dashed lines indicate the best linear fit.

Solar photovoltaic (PV) is widely celebrated within the technology literature for displaying an extremely steep experience curve (15). In addition, the comparison with space seems appropriate given solar PV research was accelerated by the race to space in the late 1950s and the need to power artificial satellites. Between 1975 and 2019, solar PV module costs dropped on average by 20.2% for each doubling of installed capacity. In 2023 dollars, the price per Watt of solar PV went from 130 USD in 1975 to 0.31 USD in 2023. Solar PV displays a faster drop in costs than any other renewable energy technology, including onshore wind, offshore wind, and concentrating solar power (27). We therefore proceeded to compare the experience curve of space launch with that of solar PV over a comparable time horizon. We merged our space dataset with data on the cost and installed capacity of solar PV (see Materials and methods). At first glance, it is evident that solar has displayed sharper drops in prices, given that the price dropped by 99.8% for solar and 85.3% for space between 1975 and 2023. In terms of Moore's Law, space displays a faster progress ratio. However, what is remarkable is that space achieved those cost reductions with a much smaller increase in cumulative scale. In other words, when we compute the experience curve based on Wright's Law for space and solar PV between 1975 and 2023, that of space results to be steeper (Fig. 3). More formally, we can reject that *β*_space_ ≥ *β*_solar_ at the 1% level under a variety of specifications (Table S3). In addition to comparing across the whole time horizon, we also ran a basic structural break test for both solar PV and space technology, as it has been shown that technology progress ratios can be variable (28). This test identified 2006 and 2010 as years in which the cost reduction of solar and space accelerated, respectively. At least for what concerns space, there are reasons to suspect that 2010 was identified because it is the year in which SpaceX's Falcon 9 was first introduced on the back of NASA's COTS program, as suggested also in the literature (11), marking a structural acceleration in the amount of payload that could be sent to space (Figs. S4 and S5) and in the technological progress ratio. We then compared experience curves only for this subsample and we could still reject that *β*_space_ ≥ *β*_solar_ at the 1% level. To put it simply, to the extent that there is an acceleration in both experience curves, space is still beating solar PV in terms of experience at comparable levels of deployment. We take this as strong evidence that the experience curve of space is quantitatively very steep.

### Historical comparison

Historical comparisons between the space economy and the roll-out of Earth-based technologies are frequent in the literature (29, 30). In line with this approach, we merged our space transport data with historical data on freight (31). We then looked at the impact on the cost of shipping wheat and cotton across the Atlantic in the aftermath of the introduction of the steam ship in 1819, which paved the way for the First Wave of Globalization (32). We find that the cost reduction of sending a kilogram to orbit observed since 1960 is faster than the fall in costs of wheat or cotton following the introduction of the steamer during the First Industrial Revolution (Fig. S6 and Table S4). In other words, in terms of Moore's Law, space has a steeper experience curve. Historical freight data loosely respects Wright's Law (*R*^2^ = 0.559). For every doubling in the cumulative amount of freight, the average cost of cotton and wheat decreased by 15.5%. However, space displayed a steeper experience curve also in terms of Wright's Law. This was true (*P* < 0.01) under a wide variety of specifications, whether considering only wheat, cotton, or the pooled sample.

Given the substantive implications that the steamer had for world trade in the 19th and early 20th centuries, we take this as suggestive evidence that the cost reductions observed in space hitherto could already pave the way for a significant expansion in the size of the space economy. It is, however, unlikely that all the possible cost reductions for space launch technology have already been achieved, especially given this has happened at relatively small levels of deployment. We therefore leverage our methodology to project the likely further reduction in the cost of accessing space over the coming years.

### Technological forecasting

**Figure 4:**
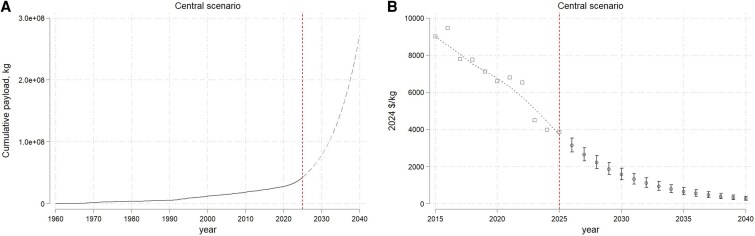
Central scenario, cumulative payload (A) and cost per kilogram of payload to LEO (B). The A chart indicates the time series of cumulative payload to orbit (solid line) and the forecasted cumulative capacity under our central scenario assumptions (dashed line). The B chart indicates the evolution of costs per kilogram of payload between 2015 and 2025, and the forecasted average costs per kilogram based on our model and central scenario assumptions. Dotted lines indicate the beginning of the forecasting horizon. Brackets indicate 95% CIs.

The literature suggests Wright's Law produces the best forecasts when trying to predict technological improvements (25, 33–35). We can therefore leverage the fact that space follows almost perfectly Wright's Law to estimate future out-of-sample launch costs under a set of alternative scaling-up scenarios. Our central scenario assumes that payload to orbit continues to grow at the average rate observed between 2010 and 2025, i.e. 13.3%. We pick 2010 given that, as discussed above, a structural break test suggests there has been an acceleration in the progress ratio of the experience curve since then (Fig. S4). Given the slope of the space experience curve, in our central scenario the average cost of bringing 1 kg of payload to orbit is expected to drop from roughly 3,800 USD today to 1,600 USD at the end of this decade, all the way to 300 USD in 2040 (Fig. 4). We stress test our results in several ways. First, we build a more conservative and a more bullish scenario for the growth of payload to orbit. These effectively use a longer (30 years) or shorter (10 years) time window when calculating the average rate that is then used to project the future growth of cumulative payload to orbit. Second, we acknowledge the arbitrary nature of our projections. As such, we deploy an entirely data-driven method of univariate time-series forecasting to project cumulative payload to orbit into the future (see Materials and methods, for more information). The scenario based on this error-trend-seasonality (ETS) exponential smoothing model results to be very conservative, with levels of cumulative payload 11% lower than our conservative scenario in 2030 and 40% lower in 2040. Nonetheless, under any of the scenarios considered, space launch costs per kilogram are expected to drop significantly vis-à-vis their current level (Fig. S7). Even under the most conservative ETS scenario, the cost of sending 1 kg of payload to orbit would be 45% lower than current levels by 2030, and 75% lower by 2040. This is intuitively the case because the experience curve for space is extremely steep, as discussed above, and has in-built decreasing returns to scale.

In order to stress test our results, we use our methodology to hindcast space launch costs. In other terms, we estimate the experience curve for space until 2015 and use Wright's Law to forecast launch costs until 2025. We then compare the actual realizations with our model-based empirical estimates. This method suggests that using a long-term 30-year trend to forecast future growth in cumulative payload, as we do in our conservative scenario, would have yielded the best predictions, all within the model's margins of error up until 2023 (Fig. S8). ETS presents the worst forecasting performance. At any rate, this robustness check suggests that our overall methodology, under all the scenarios considered, is likely to be underestimating future price reductions. This should perhaps not surprise given our model is by necessity calibrated on the past, and the space sector is currently undergoing a sharp acceleration, entering an exponential growth phase. The growth of yearly payload to orbit since 2020 has been 31%, vis-à-vis an average growth rate of 4% between 2000 and 2019. We take all this as evidence that our results should be treated at best as conservative estimates. Based on the data, we cannot discount the possibility that the average cost of sending a kilogram to orbit will fall far more quickly than we have forecasted.

## Discussion

To recapitulate, we have shown that space launch technology follows Wright's Law almost perfectly and exhibits a very fast experience curve, and that the recent phase of cost reduction reflects both genuine learning by doing within rocket families and a reallocation of payload toward cheaper launchers, with the latter dominating over the past three decades. Under a set of assumptions about the future evolution of payload to orbit, we are able to project the likely evolution of the cost of sending a kilogram to space. Our central scenario suggests costs per kilogram might more than halve between now and 2030, and drop further by four-fifths by 2040. It is important to stress that the distance–cost continuum works quite differently in space than on Earth, meaning that large amounts of energy are needed to get off our planet but once geostationary orbit is reached (about 36,000 km above Earth), the gravitational pull is 1/50th of that at sea level. From there onwards, the energy needs to reach the Moon or Mars are somewhat similar (36). In other terms, the observed and projected falling costs of exiting Earth's orbit could significantly pave the way to space colonization and economic growth well beyond simple LEO (37, 38).

It is worth comparing the quantitative estimates originating from our empirical model with facts on the ground. Our central scenario effectively implies that by 2030, humanity will have the joint capacity to send 9,100 tons per year to LEO. To put this into perspective, SpaceX alone could achieve this with 80 flights of its new super-heavy rocket Starship, which is designed to be fully reusable. In 2025 alone, SpaceX launched its partially reusable Falcon 9 rocket over 165 times.

SpaceX's Starship is likely to be a crucial further advancement in terms of lowering the cost of access to space, just as Falcon 9's partial reusability did from 2010 onwards. Starship is still in advanced prototype phase, but likely to enter into service already in late 2026. If that were the case, from day one its launch cost to payload is estimated to be around 1,000 USD/kg, given full reusability. In addition, space engineering experts expect a significant margin for rapid cost reductions from there on, as production gets standardized, possibly bringing costs below 500 USD/kg over the next few years (39). This is particularly important because, at the global level, SpaceX is currently responsible for roughly 75% of total payload sent to orbit. In other words, future cost reductions in space access are likely to rely very heavily on the capacity of SpaceX to scale up its Starship rocket. The same can be said of Blue Origin, which is currently prototyping its own super-heavy rocket New Glenn, and would have an initial estimated cost of roughly 1,500 USD/kg. In parallel, China is rapidly expanding its space program, while also prototyping reusable rockets that will likely enter the market over our forecasting horizon. All these forces could lead to a sharper fall in costs than our central scenario might suggest.

The fact that the United States and the whole world have become so reliant on a single company for cheap space access makes the task of projecting the size of the space economy particularly challenging. While costs are interesting from a technology perspective, prices are what will be charged to those wanting to access space. Economic theory suggests that a profit-maximizing (quasi-)monopolist will have a strong incentive to charge higher prices to potential clients, and some evidence already points in this direction (40). This in turn will reduce the total payload sent to orbit, and therefore affect the future evolution of average launch costs. In addition, the current climate of increasing geopolitical tensions is likely to make governments around the world wary of relying too heavily on a single American provider for space access, as this dependency could be weaponized. Effectively, this is likely to bring us back to the Cold War dynamic described in Fig. 1, where the dispersion of costs might widen once again, as different countries pursue strategic autonomy even if this means using more expensive home-based rocket technology. This is particularly problematic because our Bennett decomposition shows how cost reductions are predominantly a result of reallocation of payload to cheaper rockets. Geopolitical tension can therefore exert a significant slowing effect on the future fall of average launch costs.

Finally, and relatedly, we note the difficulty of engaging in forecasting at times of structural breaks. Just above we were noting a structural shift in the geopolitical climate, but the problem extends further. As our descriptive statistics suggest, the space sector seems to be experiencing a sharp transformation. More objects were launched into space in the last 5 years than in the preceding five decades. Around the time of these structural breaks in trends, forecasting is bound to be challenging, irrespective of the model used. This is because models are necessarily calibrated on past data. Throughout the paper, we have highlighted how this could lead to an upward bias in our future cost estimates. At the same time, other dynamics beyond our model could intrude. For instance, the current exponential growth in space-based objects could lead to a worsening of the issue of space debris, which could escalate into a problem for the use of LEO and force a slowdown in the current rate of growth of payload to orbit (41).

We consider this paper a first attempt to scratch the surface of the future evolution of the space economy. As such, several avenues for future research are available, including by leveraging further the dataset made available by this paper. We highlight one in particular. With our focus on technology and costs, we have effectively zoomed in on the supply side of the space economy, and launch technology more specifically. Future work could incorporate a model for the demand side, to complement our analysis and move from a cost perspective to price estimations. This would allow a better gauge of the actual size of the expanding space economy.

## Materials and methods

Here, we describe the data collection method, the comparative analysis performed and the model details used for the projection of space launch costs into the future. All data and STATA code used in the analysis are available in the Supplementary material.

### Data

We have assembled an unbalanced panel dataset of all space launches from 1960 to 2025 for the following countries: Australia, Brazil, India, Israel, Iran, Japan, Republic of Korea, China, Russia, Ukraine, the United States, and Europe. The latter is a combination of launches made under the umbrella of the European Space Agency through the Ariane program, starting in 1979, and individual European countries (France, Italy, Germany, and the United Kingdom). Costs are estimated based on the so-called “unit flyaway cost,” including all direct and indirect manufacturing costs and their associated overhead plus recurring engineering, sustaining tooling, and quality control but crucially excluding the costs associated with researching and developing the vehicle design before manufacturing individual, launch-ready vehicles. Several data sources, including government reports, academic papers, and public documentation, were used to populate the dataset, and for each rocket configuration they are reported in the dataset (42, 43). The most recurrent data source is “Encyclopedia Astronautica” (44), especially for older rockets, while for more modern ones cost estimates are widely available via public online sources. Data were also cross-checked with a variety of other miscellaneous sources (45–49). Given the wide variety of rocket configurations within the same family, occasionally, to fill in data gaps, cost information on the closest rocket of the same family was used, as detailed in the notes section of the dataset in the Supplementary material. While different rockets are designed to bring payload to different altitudes, for example Geostationary Transfer Orbit, all our data are standardized to LEO, using standard engineering conversion coefficients, in line with standard practice in the literature (22). In addition, all costs are converted to 2024 USD. In order to perform the Bennett decomposition, rocket configurations were associated with their rocket family rather than manufacturer information, as the latter are in constant evolution due to mergers, acquisitions, and joint ventures. No outlier was arbitrarily excluded from the dataset or to perform any of the analyses in the paper. In order to ensure full replicability, in the dataset included in the Supplementary Material, we provide the data source used for each rocket configuration, and in the notes section, we specify whether imputations were made, and what engineering coefficients were used to standardize all figures to LEO equivalent.

### Wright's Law estimation

We ran a basic regression of cost over cumulative payload in log terms over the full sample. We then tested different time frames, and in particular noted how the *R*^2^ became higher in the post-Cold War era and was very high if we used the subsample 2000–2025 (Fig. 2). Augmented Dickey–Fuller tests show that cumulative payload (in logs) is stationary (*I*(0), MacKinnon *P* = 0.003) in levels with a deterministic trend, while average costs (in logs) is nonstationary (*I*(1), MacKinnon *P* = 0.344). Because the regressor is *I*(0), the regression of cost on cumulative payload in nondifferenced log form does not raise spurious-regression concerns. Following standard practice in experience-curve estimation (15, 25), we therefore estimate the model in log levels (i.e. without differencing) and report Newey–West HAC SEs (with two lags) to ensure valid inference under serial correlation. Inference is robust across multiple corrections for serial correlation. Specifically, we estimated also an AR(1) and AR(2) Prais–Winsten models. Coefficients and significance levels are unchanged, indicating that autocorrelation does not materially affect the results. After running the basic experience curve regression, we ran a single structural break test. This was conducted via a supremum of Wald statistics test, verifying the stability of coefficients throughout time, and 2010 was identified as a structural break year (*P* = 0.000). We took this as evidence that the experience curve of space launch technology might have accelerated since 2010, i.e. since the introduction of SpaceX's Falcon 9 rocket on the back of NASA's COTS program. All these results are reported in Table S1. In addition, we estimated separately the learning curve of space launches within rocket families. To do so, we ran a set of panel regressions with fixed effects and clustered SEs, with average costs as a function of cumulative payload within a family, all in logs. The R^2^ remains <0.5 in all specifications, suggesting Wright's Law is a better approximation for the aggregate experience curve than for the microlevel learning curve of space launch technology. Nonetheless, the coefficient was negative and significant in all specifications, including with full year fixed effects, which should correct for homogenous shocks to all rocket families, such as changes in market structure. All these results are reported in Table S2.

### Comparative analysis

In order to perform our comparative analysis of space experience curve with solar PV, we downloaded data for solar PV modules cost and cumulative capacity between 1975 and 2023 from “Our World in Data” (50). In order to merge it with our space dataset, given the differences in the unit of measurement and technology, we indexed both unit costs and cumulative capacity, for space and solar PV, setting 1975 = 100. We then compared the experience curve by plotting the log of cost and log cumulative payload (Fig. 3), and ran a set of regressions to compare space and solar PV (Table S3). Driscoll–Kraay SEs were used to correct for autocorrelation and potential heteroskedasticity in panel setting. In Table S3, model (1) shows how space has reduced its cost more slowly than solar when considering Moore's Law. From models (2)–(4), we consider Wright's Law. Model (2) considers the whole sample, 1975–2023. Model (3) compares the faster experience curve that space has experienced since 2010 (based on the structural break test performed previously) with the overall experience curve of solar PV. Finally, we performed a similar structural break test for solar PV's experience curve, identifying 2006 as a year of acceleration in the progress ratio. In model (4), we therefore compare only space since 2010 with solar PV since 2006.

In order to perform our historical comparative analysis, we downloaded historical trade data on freight imports to the United Kingdom from the United States from 1800 to 1938 (31). Given the data were highly volatile, we took 5-year rolling averages of price levels of freight for both wheat and cotton. We then indexed the data setting 1818 = 100, based on the fact that the SS Savannah was the first steam ship to cross the Atlantic from May to June 1819 (32). Similarly for comparison, we performed a 5-year rolling average on our space cost data and set 1964 = 100. This is because our dataset starts in 1960 and we needed five observations for the first 5-year rolling average point. For what concerns historical quantity data, we used an index of world export from the same database and rebased it so that 1818 = 100. Similarly, we indexed our cumulative payload to orbit data so that 1964 = 100. We then performed a set of standard regressions, reported in Table S4. Specifications (1) and (2) show how well freight data respects Moore's and Wright's Law. Based on the *R*^2^, Wright's Law seems a better fit to the data. Model (3) shows that space experiences a faster experience curve than historical freight under Moore's Law. Model (4) shows that space experiences a faster experience curve than historical freight under Wright's Law. Models (5) and (6) show this remains the case when excluding cotton or wheat. No space-specific constant was introduced, irrespective of having an interaction effect, given the data are all indexed to start at the same level of 100.

### Out of sample predictions

In order to perform our predictions out of sample, we made use of the experience coefficient derived from regressing cost on cumulative payload in logs. Based on the finding that 2010 marked a structural break and that there are reasons to believe the progress ratio for space has accelerated since then, for our calibration we used this faster experience coefficient, as estimated in Model (5) in Table S1. For our scenario-based analysis, we produced three sets of scenarios for the evolution of payload to orbit between 2025 and 2040. For the central scenario, we assumed payload to orbit would continue to grow at 13% per annum, which is the average rate at which it expanded over the period 2010–2025. For the conservative scenario, we assumed payload to orbit would continue to grow at 7.5% per annum, which is the average rate at which it expanded over the period 1995–2025. For the bullish scenario, we assumed payload to orbit would continue to grow at 21% per annum, which is the average rate at which it expanded over the period 2016–2025. For our data-driven scenario, we deploy a basic ETS univariate time-series forecasting method. Specifically, we used a Holt–Winters Exponential Smoothing function, with (0.9, 0.9) as calibrating parameters, giving therefore a stronger weight to the last observations. In order to check the robustness of our forecasting methodology, we performed a hindcasting exercise, in line with standard practice in the literature (15). Effectively, we did so by dropping all observations after 2015 from our dataset and rerunning the commands for the scenario-based and data-driven approach, using them to forecast up until 2025. We then compare the out-of-sample forecasts with the actual realizations of costs per kilogram (Fig. S8).

## Supplementary Material

pgag217_Supplementary_Data

## Data Availability

The authors confirm that the data supporting the findings of this study are available in Data Files S1–S3.

## Notes

a These results are in line with recent model-based literature on market structure and innovation in the space launch industry (22) but represent a useful complement as the lack of empirical data forced modelers to make parametric assumptions on the degree of within-family learning by doing.

b Several other models of experience curves have been postulated, such as Goddard’s Law or Nordhaus’s model, but are generally considered less useful, especially for technological forecasting (33).

## References

[1] Jaffe AB, Fogarty MS, Banks BA. 1998. Evidence from patents and patent citations on the impact of NASA and other federal labs on commercial innovation. J Ind Econ. 46:183–205.

[2] Corrado L, Grassi S, Paolillo A, Silgado-Gómez E. 2023. The macroeconomic spillovers from space activity. Proc Natl Acad Sci U S A. 120:e2221342120. 37844249 10.1073/pnas.2221342120 PMC10614782

[3] Space Foundation. 2025. The Space Report 2025 Q2. Space Foundation. [accessed 2026 Jun]. https://www.spacefoundation.org/2025/07/22/the-space-report-2025-q2/

[4] Manson K, Shepherd C. US Military officials eye new generation of space weapons. Financ Times, 2020.

[5] Stockdale P, Aughenbaugh S, Boensch NJ. 2018. Low-cost access to space: military opportunities and challenges. Defense Horizons 5. https://digitalcommons.ndu.edu/defense-horizons/5

[6] Terzi A, Nicoli F. 2024. Space possibilities for our grandchildren: current and future economic uses of space. Eur Econ Discuss Pap. 211:1–34.

[7] Corrado L, Cropper M, Rao A. 2023. Space exploration and economic growth: new issues and horizons. Proc Natl Acad Sci U S A. 120:e2221341120. 37844229 10.1073/pnas.2221341120 PMC10614830

[8] Sandler T, Schulze W. 1981. The economics of outer space. Nat Resour J. 21:371–393.

[9] Weinzierl M . 2018. Space, the final economic frontier. J Econ Perspect. 32:173–192.

[10] Koelle DE . 1989. Launch cost analyses for reusable space transportation systems (Sänger II). Acta Astronaut. 19:191–197.

[11] Jones HW . 2018. The recent large reduction in space launch cost. NASA Technical Reports Server, Report No. ARC-E-DAA-TN56851. https://ntrs.nasa.gov/citations/20200001093

[12] Williams I, Dahlgren M, Roberts TG, Karako T. *Boost-phase missile defense: interrogating the assumptions*. Center for Strategic and International Studies, 2022. https://www.csis.org/analysis/boost-phase-missile-defense

[13] Adilov N, Alexander P, Cunningham B, Albertson N. 2022. An analysis of launch cost reductions for low Earth orbit satellites. Econ Bull. 42:1561–1574.

[14] Nicoli F, Sekut K, Porcaro G. 2023. Can Europe make its space launch industry competitive? Analysis 21/2023, Bruegel. https://www.bruegel.org/analysis/can-europe-make-its-space-launch-industry-competitive

[15] Farmer JD, Lafond F. 2016. How predictable is technological progress? Res Policy. 45:647–665.

[16] Thompson P . 2012. The relationship between unit cost and cumulative quantity and the evidence for organizational learning-by-doing. J Econ Perspect. 26:203–224.

[17] Kulu E . In-space manufacturing—2022 industry survey and commercial landscape in proceedings of the international astronautical congress. International Astronautical Federation, 2022.

[18] Hirschberg C, Kulish I, Rozenkopf I, Sodge T. The potential of microgravity: how companies across sectors can venture into space. McKinsey Co, 2022.

[19] Sharma A, et al 2022. Biomanufacturing in low Earth orbit for regenerative medicine. Stem Cell Reports. 17:1–13. 34971562 10.1016/j.stemcr.2021.12.001 PMC8758939

[20] Kantor S, Whalley A. 2025. Moonshot: public R&D and growth. Am Econ Rev. 115:2891–2925.

[21] Juhász R, Squicciarini MP, Voigtländer N. 2024. Technology adoption and productivity growth: evidence from industrialization in France. J Polit Econ. 132(10):3215–3259. 10.1086/730205

[22] Su R, Yang C, Sweeting A. 2026. Competition, procurement and learning-by-doing in the space launch industry. NBER Working Paper 34766. 10.3386/w34766

[23] Klepper S . 1996. Entry, exit, growth, and innovation over the product life cycle. Am Econ Rev. 86:562–583.

[24] Wright TP . 1936. Factors affecting the cost of airplanes. J Aeronaut Sci. 3:122–128.

[25] Nagy B, Farmer JD, Bui QM, Trancik JE. 2013. Statistical basis for predicting technological progress. PLoS One. 8:e52669. 23468837 10.1371/journal.pone.0052669 PMC3585312

[26] Moore G . 1965. Cramming more components onto integrated circuits. Electronics (Basel). 38:19.

[27] IRENA . *Renewable power generation costs in 2019*. International Renewable Energy Agency, Abu Dhabi, 2020.

[28] Carlino A, et al 2025. Variability of technology learning rates. Adv Appl Energy. 20:100252.

[29] Fogel RW . 1966. Railroads as an analogy to the space effort: some economic aspects. Econ J. 76:16.

[30] Launius RD . Historical analogs for the stimulation of space commerce. NASA, 2014.

[31] Federico G, Tena-Junguito A. 2019. World trade, 1800–1938: a new synthesis. Rev Hist Econ—J Iber Lat Am Econ Hist. 37:9–41.

[32] Smil V . Energy and civilization: a history. MIT Press, 2017.

[33] Meng J, Way R, Verdolini E, Anadon LD. 2021. Comparing expert elicitation and model-based probabilistic technology cost forecasts for the energy transition. Proc Natl Acad Sci U S A. 118:e1917165118. 34183405 10.1073/pnas.1917165118 PMC8271727

[34] Way R, Ives MC, Mealy P, Farmer JD. 2022. Empirically grounded technology forecasts and the energy transition. Joule. 6:2057–2082.

[35] Alberth S . 2008. Forecasting technology costs via the experience curve—Myth or magic? Technol Forecast Soc Change. 75:952–983.

[36] Pelton JE . The new gold rush: the riches of space beckon. Springer US, 2016.

[37] Weinzierl M . 2023. Expanding economic activity in space may offer a solution to secular stagnation. Proc Natl Acad Sci U S A. 120:e2221347120. 37844227 10.1073/pnas.2221347120 PMC10614598

[38] Nicoli F, Terzi A. Economic growth in space. In: Davies R, Francois J, Polanco R, editors. Regulating space-based commerce: insights from economics and international economic law. Hart Publishing, 2026. https://www.bloomsbury.com/uk/regulating-spacebased-commerce-9781509992065/

[39] Poling A . 2023. T minus six seconds: starship (and humanity's) next major step into space. Georg Secur Stud Rev. https://gssr.georgetown.edu/the-forum/topics/technology/t-minus-six-seconds-starship-and-humanitys-next-major-step-into-space/

[40] Kim MJ . 2025. Counting stars and costs: an empirical examination of space launch cost trend at NASA. Acta Astronaut. 232:633–639.

[41] Adilov N, Alexander PJ, Cunningham BM. 2015. An economic analysis of Earth orbit pollution. Environ Resour Econ. 60:81–98.

[42] Eggermont J . 2022. The European space sector's position on the launch market in the NewSpace age. https://libcatalog.ugent.be/nde/fulldisplay?context=L&vid=32RUG_INST:32RUG_INST&search_scope=MyInst_and_CI&docid=alma990031176580409161

[43] Isakowitz SJ . International reference guide to space launch systems. AIAA, 1991.

[44] Wade M . 1999. Encyclopedia Astronautica. (April 10, 2025). http://www.astronautix.com/

[45] Krebs GD . 1996. Gunter's Space Page. (April 10, 2025). https://space.skyrocket.de/index.html

[46] Bonnett EW . A cost history of the Thor-Delta launch vehicle family. International Astronautical Federation, 25th International Astronautical Congress; Amsterdam; 1974, p. 36.

[47] Futron Corporation . 2002. Space transportation costs: trends in price per pound to orbit 1990–2000. Futron Corporation. https://www.yumpu.com/en/document/view/36996100/space-transportation-costs-trends-in-price-per-pound-to-orbit-

[48] Wertz JR, Larson WJ. Reducing space mission cost. Springer, 1996.

[49] Roberts TG . 2022. Space launch to low earth orbit: how much does it cost? CSIS. (April 11, 2025). https://aerospace.csis.org/data/space-launch-to-low-earth-orbit-how-much-does-it-cost/

[50] Ritchie H, Rosado P, Roser M. 2023. Solar photovoltaic module price. Our World Data. (April 10, 2025). https://ourworldindata.org/grapher/solar-pv-prices

