# Supplementary material for: From Sputnik to Starship: Estimating the experience curve of space launch technology
Source: PNAS Nexus. 2026 Jul 14;5(7):pgag217. doi: 10.1093/pnasnexus/pgag217 (PMC13366537; doi:10.1093/pnasnexus/pgag217)
Supplement: pgag217_Supplementary_Data [file pgag217_supplementary_data.zip › PNASNEXUS-PNASNEXUS-2026-00061-TRR-s01.pdf]

PNAS nexus

Supplementary Information for
From Sputnik to Starship: Estimating the experience curve of space launch technology.

Alessio Terzi* and Francesco Nicoli

*Corresponding author. Email: at2113@cam.ac.uk

This PDF file includes:

Figs. S1 to S8
Tables S1 to S4

Other supplementary materials for this manuscript include the following:

Data S1 to S3

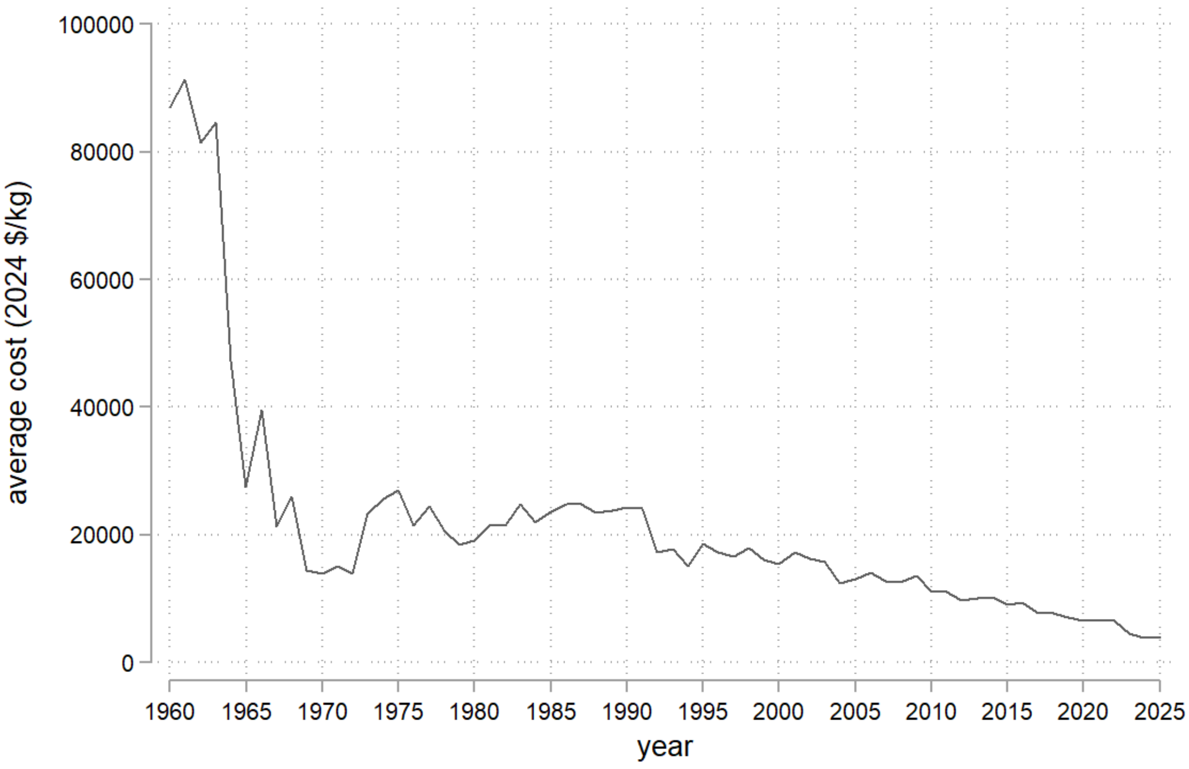

Fig. S1.

Average launch cost, world. This chart represents the time series estimate of the average cost of launching a kilogram of payload into Low Earth Orbit between 1960 and 2025, expressed in 2024 USD.

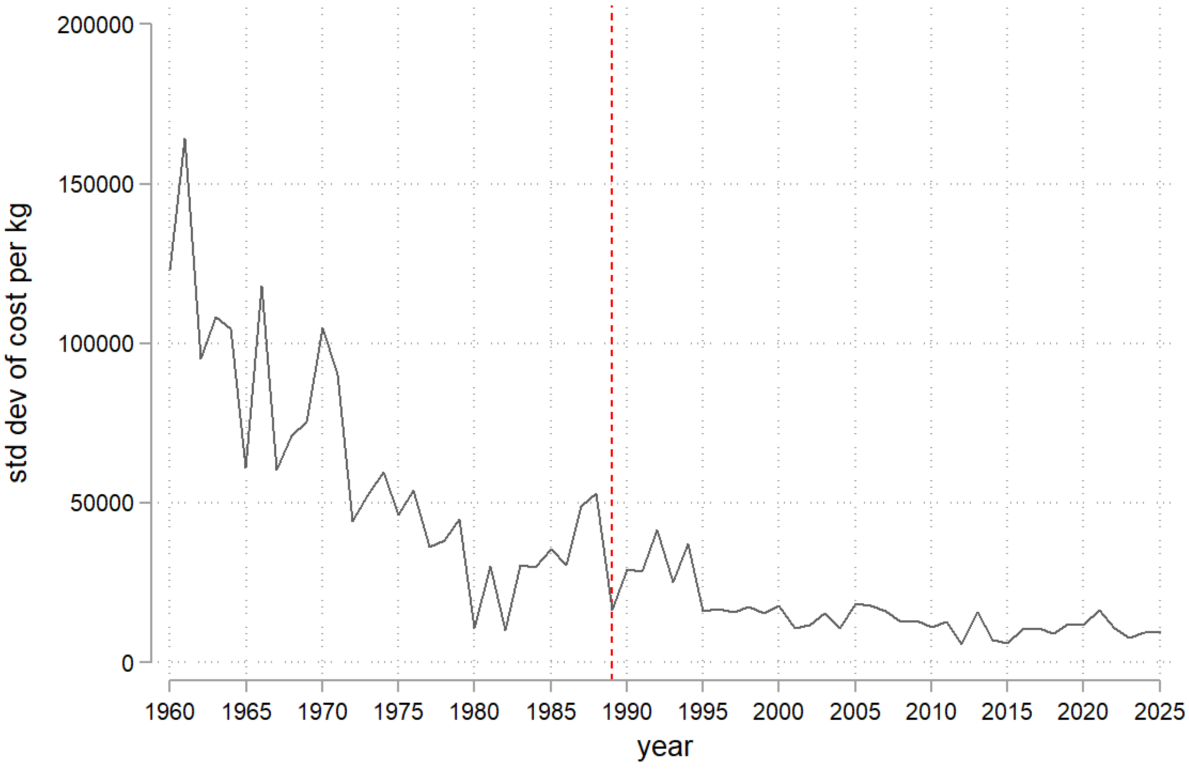

Fig. S2.

Standard deviation of launch costs. The chart represents the time series estimate of the standard deviation of the cost of sending a kilogram of payload to Low Earth Orbit, expressed in 2024 USD. The chart was produced excluding five outliers (costs of more than 1 million USD per kg). The red dashed line indicates the end of the Cold War.

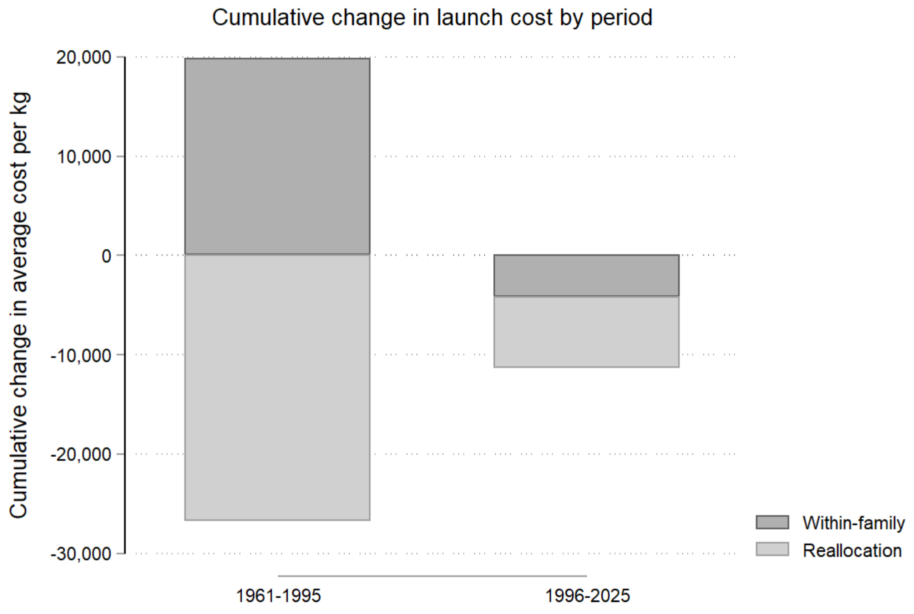

Fig. S3.

Bennett decomposition of changes in launch costs. The figure decomposes aggregate cost change into a within-family and a reallocation component, split between two periods: pre-1995 and post-1995.

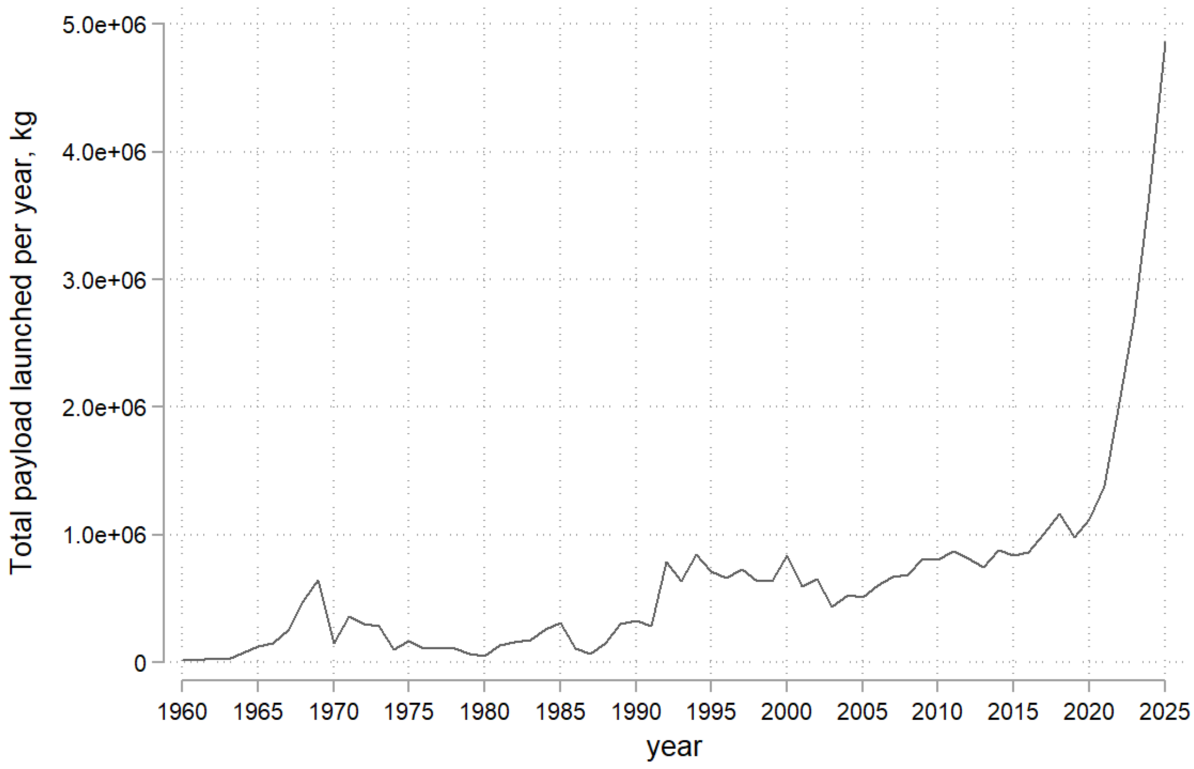

Fig. S4.

Total payload launched to space per year, world. The chart displays the total payload launched across all launchers between 1960 and 2025, converted in LEO equivalent.

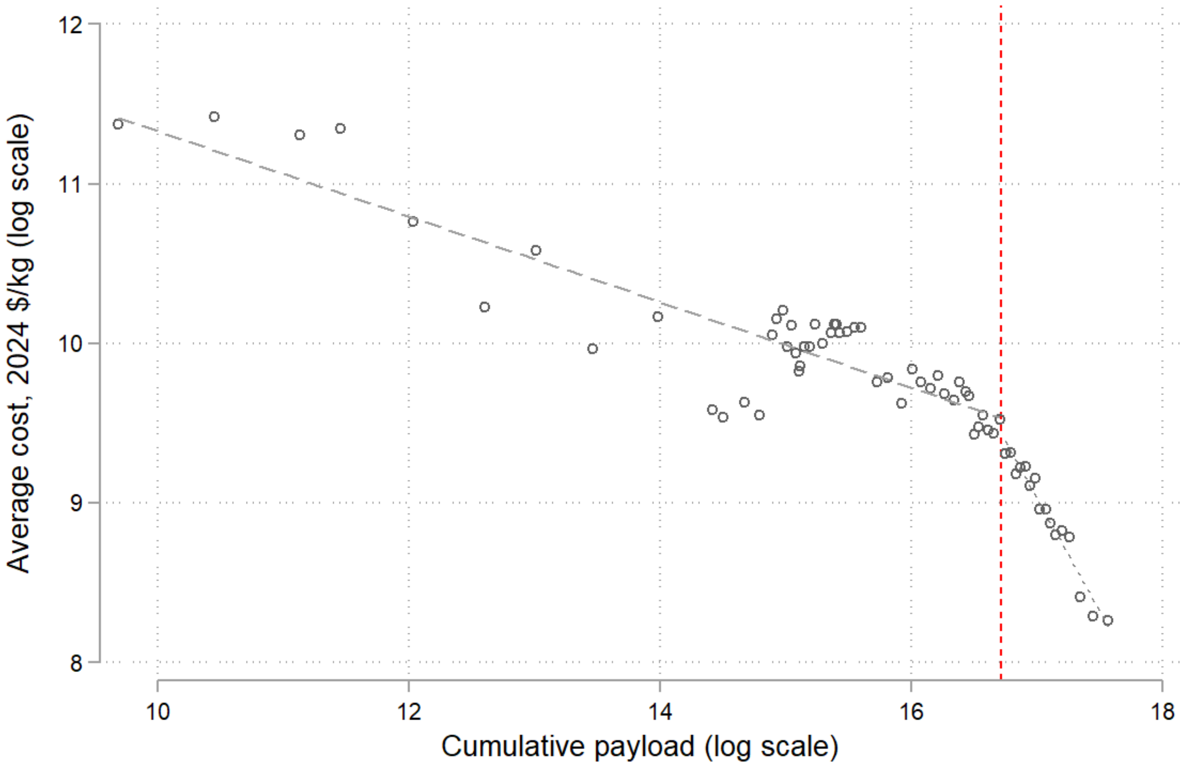

Fig. S5.
Experience curve for space before and after 2010. The chart represents the average cost of launching one kilogram of payload to Low Earth Orbit on the y-axis and the cumulative payload to orbit on the x-axis, both in log scale, between 1960 and 2024. The red-dashed line indicates 2010. The dashed grey and dashed black lines indicate the best linear fit before and after 2010. 2010 was identified as a structural break in the stability of the regression coefficient by performing a test based on the supremum of Wald statistics test.

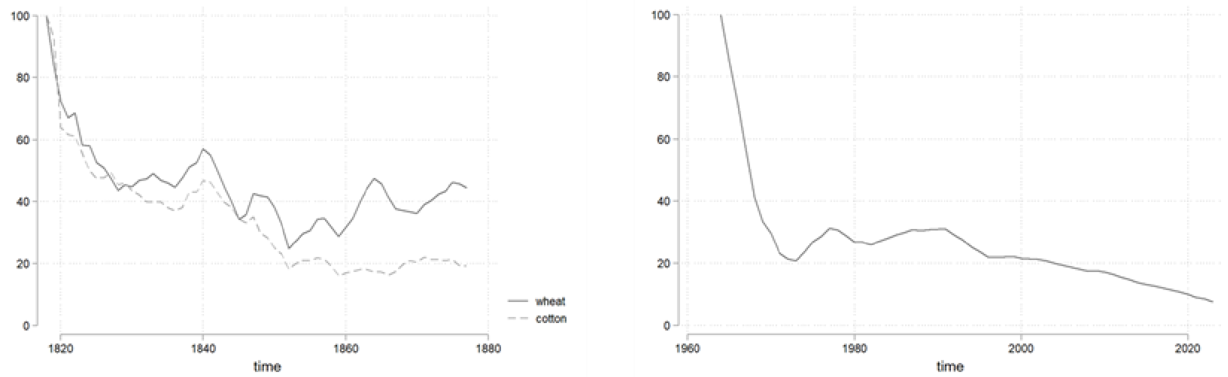

Fig. S6.
Evolution in the average costs of cotton and wheat freight, and space technology.
Indexed 5-year rolling average of costs for cotton, wheat (LHS) and space (RHS) over a comparative time horizon. For cotton and wheat, 1818=100. For space, 1964=100.

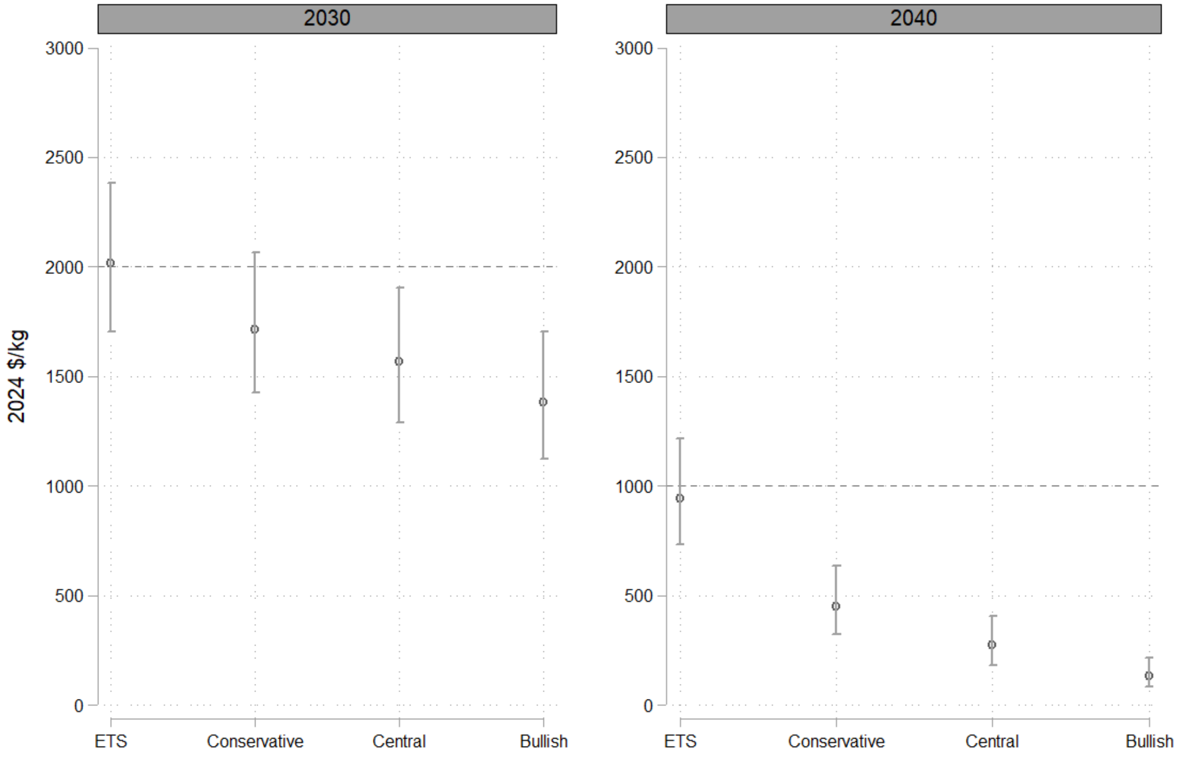

Fig. S7. Out-of-sample projections of average launch cost reduction by 2030 (LHS) and 2040 (RHS) under a set of alternative scenarios. ETS is a data-driven time-series scenario. Conservative, central and bullish are different realisations of our baseline model under a different set of assumptions regarding the growth of payload to orbit. Brackets indicate 95% confidence intervals. Dashed grey lines indicate the 2000 USD and 1000 USD benchmark level.

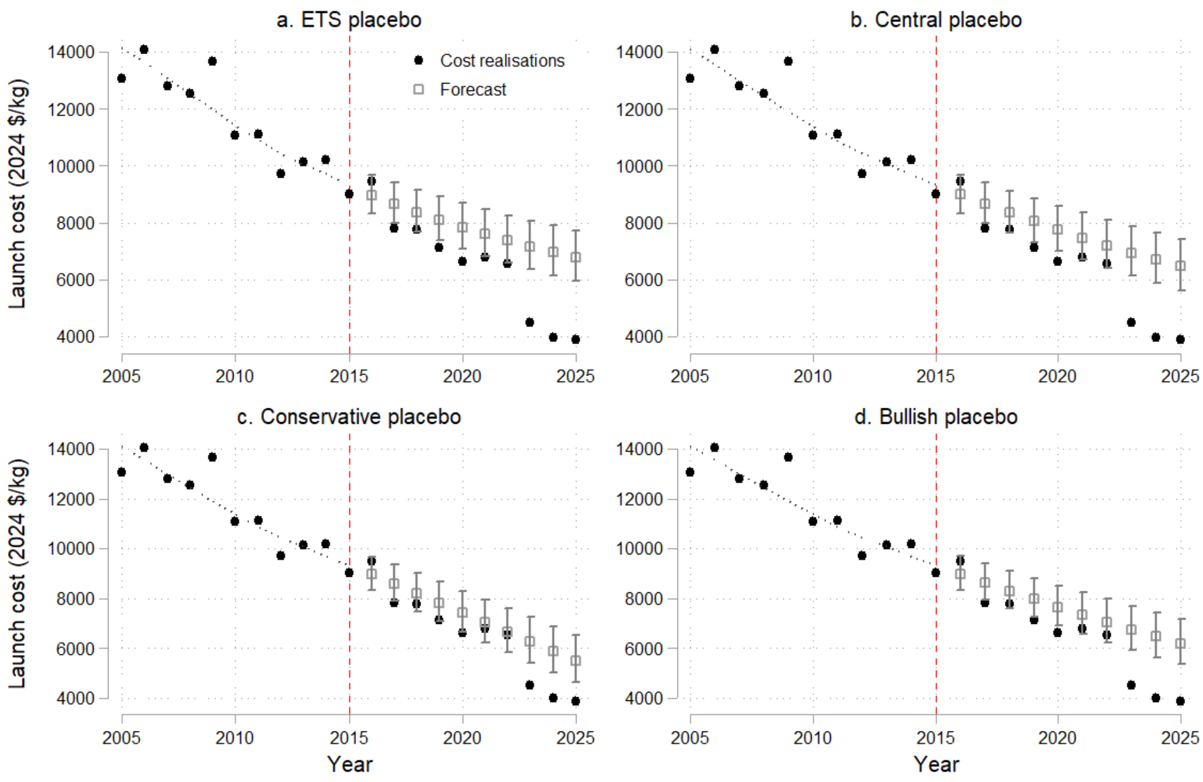

Fig. S8. Hind-casting of average launch costs under alternative projection methods. ETS is a data-driven time-series scenario. Conservative, central and bullish are different realisations of our baseline model under a different set of assumptions regarding the growth of payload to orbit. Black dots indicate cost realisations. Gray squares indicate model-based projections. Brackets indicate 95% confidence intervals. Red dashed line indicates 2015.

	(1)	(2)	(3)	(4)	(5)	(6)	(7)
	ln(costs)	ln(costs)	ln(costs)	ln(costs)	ln(costs)	ln(costs)	ln(costs)
ln(cumulative payload)	-.343***	-.276***	-.836***	-1.213***	-1.412***	-.343***	-.343***
	(.076)	(.030)	(.086)	(.071)	(.083)	(.028)	(.031)
constant	15.055***	14.110***	23.234***	29.619***	33.039***	15.055***	15.055***
	(.417)	(.430)	(1.426)	(1.201)	(1.415)	(.423)	(.467)
Estimation model	OLS	OLS	OLS	OLS	OLS	Prais–Winsten AR(1)	Prais–Winsten AR(2)
Standard errors	Newey-West HAC	Newey-West HAC	Newey-West HAC	Newey-West HAC	Newey-West HAC	GLS	GLS
Cold war era only	×	✓	×	×	×	×	×
Post Cold War era only	×	×	✓	×	×	×	×
Data since 2000 only	×	×	×	✓	×	×	×
Falcon9 era only	×	×	×	×	✓	×	×
Sample size	66	30	36	26	16	66	66
R^2	0.791	0.736	0.878	0.953	0.954	-	-

Note: Standard errors in parentheses. * p<0.1; ** p<0.05; *** p<0.01

Table S1.
Estimation of the rate of technological experience (Wright's Law) for space launches under different modelling assumptions and data samples. Model (**1**) is the baseline estimation of the cost reduction under a standard OLS model for the full sample using Newey-West HAC standard errors. Models (**2**)-(**5**) are estimated in the same way, but for specific sub-samples. Models (**6**)-(**7**) are estimations based on a Prais–Winsten autoregressive model of order 1 and 2, respectively.

	(1)	(2)	(3)	(4)	(5)
	ln(costs)	ln(costs)	ln(costs)	ln(costs)	ln(costs)
ln(cumulative payload)	-0.190***	-.201***	-.118**	-.095*	-.119**
	(.035)	(.036)	(.053)	(.055)	(.049)
year			-.012**		
			(.004)		
constant	12.145***	12.317***	34.497***	11.664***	11.554***
	(.408)	(.442)	(8.468)	(.518)	(.596)
Family FE	✓	✓	✓	✓	✓
Year FE	×	×	×	✓	✓
Only families with 10+ rocket-years	×	✓	✓	✓	✓
Post Cold War era only	×	×	×	×	✓
Sample size	747	638	638	638	485
R^2	0.350	0.358	0.482	0.486	0.277

Note: Clustered standard errors (at family level) in parentheses. * p<0.1; ** p<0.05; *** p<0.01

Table S2.

Estimation of the rate of technological learning (Wright's Law) for space launches under different modelling assumptions and data samples. Model (**1**) is a basic estimation of the learning rate of space launch within rocket families under a standard panel regression, with clustered standard errors at family level. Model (**2**) includes only rocket families with at least 10 observations, to allow for learning-by-doing. Model (**3**) includes a time trend. Model (**4**) includes full family and year fixed effects, while Model (**5**) is restricted to the post-Cold-War sub-sample.

	(1)	(3)	(4)	(5)
	costs	ln(costs)	ln(costs)	ln(costs)
year	-1.228***			
	(.140)			
space x year	.024***			
	(.003)			
ln(scale)		-.388***	-.388***	-.578***
		(.014)	(.014)	(.023)
space x ln(scale)		-.172**	-1.469***	-1.279***
		(.073)	(.138)	(.166)
space (dummy=1)		.923***	9.327***	6.025***
		(.379)	(.907)	(1.272)
constant	2467.272***	6.303***	6.303***	9.605***
	(281.264)	(.140)	(.141)	(.410)
Falcon9 era only	×	×	✓	✓
Solar post-2006 only	×	×	×	✓
Sample size	98	98	63	32
R^2	0.857	0.985	0.984	0.997

Note: Driscoll-Kraay standard errors in parentheses. * p<0.1; ** p<0.05; *** p<0.01

Table S3.

Comparison of experience curves between space and solar PV. Model (**1**) is based on Moore's Law and is run in levels. Model (**2**)-(**4**) are based on Wright's Law and are in log. Model (**2**) considers the whole sample, 1975-2023. Model (**3**) compares the faster experience curve that space has experienced since 2010 (based on a structural break test) with the overall experience curve of solar PV. Model (**4**) compares only space since 2010 with solar PV since 2006 (identified as a faster experience curve based on a structural break test).

	(1)	(2)	(3)	(4)	(5)	(6)
	ln(costs)	ln(costs)	ln(costs)	ln(costs)	ln(costs)	ln(costs)
time	-.017***		-.015***			
	(.003)		(.003)			
ln(cumulative quantity)		-.244***		-.270***	-.221***	-.347***
		(.020)		(.015)	(.023)	(.015)
space x ln(cumulative quantity)				-.060***	-.082***	-.037***
				(.009)	(.011)	(.008)
space x time			-.013***			
			(.002)			
constant	4.112***	5.636***	4.035***	5.861***	5.645***	6.322***
	(.086)	(.150)	(.104)	(.122)	(.196)	(.131)
Space	×	×	✓	✓	✓	✓
Cotton	✓	✓	✓	✓	×	✓
Wheat	✓	✓	✓	✓	✓	×
Sample size	120	120	180	180	120	120
R^2	0.491	0.559	0.668	0.734	0.835	0.871

Note: Driscoll-Kraay standard errors in parentheses. * p<0.1; ** p<0.05; *** p<0.01

Table S4.

Comparison between space launch cost reductions and historical reduction in cotton and wheat freight. Models (**1**)-(**2**) verify how well freight data respects Moore's and Wright's Law. Model (**3**) shows that space has a faster experience curve than historical freight under Moore's Law. Model (**4**) shows that space has a faster experience curve than historical freight under Wright's Law. Models (**5**)-(**6**) show this remains the case when excluding cotton or wheat.

Data S1. (separate file)

This file contains the raw data of all rocket launches, in .xlsx format.

Data S2. (separate file)

This file contains the data derived from S1, aggregating by year, in .xls format. This constitutes the basis for the estimations of the experience curve for space technology and forecasts of average launch costs.

Data S3. (separate file)

This .txt file contains the STATA code for the estimation of the experience curve of space launch technology, and for the forecasts of launch costs.
